# Corrosion-Induced Degradation Mechanisms and Bond–Slip Relationship of CFRP–Steel-Bonded Interfaces

**DOI:** 10.3390/ma19030511

**Published:** 2026-01-27

**Authors:** Yangzhe Yu, Da Li, Li He, Lik-Ho Tam, Zhenzhou Wang, Chao Wu

**Affiliations:** 1 CNOOC Research Institute Ltd., Beijing 100028, China; yuyzh12@cnooc.com.cn (Y.Y.);; 2 Department of Environmental Science and Engineering, Beijing Technology and Business University, Beijing 100048, China; 3 School of Transportation Science and Engineering, Beihang University, 37 Xueyuan Road, Beijing 100191, China; 4 Institute of Cryogenics, Energy Technology Group, Highfield Campus, University of Southampton, Southampton SO17 1BJ, UK; 5 Department of Civil Environmental Engineering, Imperial College London, South Kensington, London SW7 2AZ, UK

**Keywords:** CFRP–steel bonded joints, corrosion damage, bond–slip behaviour, effective bond length, glass fibre sheet

## Abstract

Carbon fibre-reinforced polymer (CFRP) bonded steel structures are increasingly adopted in offshore floating structures, yet their interfacial performance is highly susceptible to corrosion in marine environments. Corrosion-induced degradation of the CFRP–steel interface can significantly affect load transfer mechanisms and long-term structural reliability. This paper reports an experimental study on corrosion-induced degradation mechanisms and bond–slip behaviour of CFRP–steel double-strap joints. Controlled corrosion damage was generated using an accelerated electrochemical technique calibrated to ISO 9223 corrosivity categories. Tension tests were performed to examine the effects of corrosion degree, CFRP bond length, and the inclusion of glass fibre sheets (GFS) in the adhesive layer on failure modes, ultimate load capacity, and effective bond length. Digital image correlation (DIC) was employed to obtain strain distributions along the CFRP plates and to establish a bond–slip model for corroded interfaces. The results indicate that corrosion promotes a transition from CFRP delamination to steel–adhesive interface debonding, reduces interfacial shear strength to 17.52 MPa and fracture energy to 5.49 N/mm, and increases the effective bond length to 130 mm. Incorporating GFS mitigates corrosion-induced bond degradation and enhances joint performance. The proposed bond–slip model provides a basis for more reliable durability assessment and design of bonded joints in corrosive environments.

## 1. Introduction

Offshore floating structures—including floating offshore wind turbines, wave-energy converters, and deep-water production platforms—are increasingly deployed in harsh marine environments and at greater water depths, driven by the rapid expansion of offshore renewable energy and deep-water developments [1]. In this context, CFRP bonded steel structures offer a promising solution for offshore applications. Compared with conventional welding or mechanical fastening, CFRP bonding enables efficient strengthening and repair with minimal weight addition, reduced stress concentration, and improved fatigue and durability performance [2,3,4,5]. These advantages make CFRP–steel-bonded systems well-suited for floating offshore structures, where accessibility is limited and long-term structural reliability is essential.

The bond behaviour of CFRP–steel joints play a critical role in ensuring the structural integrity and long-term reliability of CFRP-strengthened components. Extensive studies have demonstrated that the bond performance is influenced by various factors, such as the mechanical properties of CFRP laminates and adhesives [6,7,8], types of loading [9,10,11], and service conditions [12,13,14,15]. In offshore structures, bonded joints are commonly subjected to combined static, fatigue, and environmental actions throughout their service life. Therefore, understanding the degradation mechanisms of CFRP–steel-bonded interfaces under harsh marine environments is of particular importance for the safe and durable design of deep-sea offshore floating structures.

Among the environmental factors affecting offshore floating structures, corrosion damage induced by the marine environment is one of the most critical and inevitable challenges. Offshore steel components are continuously exposed to seawater, salt spray, high humidity, and cyclic wet–dry conditions, which can significantly accelerate corrosion processes. Corrosion of steel leads to thickness loss, deterioration of mechanical properties, and the formation of corrosion pits and cracks [16,17]. Meanwhile, moisture ingress into the adhesive layer may cause plasticization, micro-cracking, and hygroscopic swelling of epoxy adhesives, resulting in a reduction in adhesive strength and degradation of the steel–adhesive interface [18,19]. Furthermore, when CFRP materials are directly bonded to steel in the presence of an electrolyte such as seawater, galvanic corrosion may occur due to the difference in electrochemical potential between CFRP and steel [20]. These coupled deterioration mechanisms make CFRP–steel-bonded joints in offshore structures particularly vulnerable to corrosion-induced bond degradation.

Although considerable research has been conducted on the durability of CFRP–steel-bonded joints under marine exposure and accelerated corrosion conditions, several critical issues remain insufficiently addressed. First, most existing studies focus on global performance, such as ultimate load capacity or fatigue life, while the local interfacial bond–slip behaviour under corrosion damage has received limited attention. The bond–slip relationship is fundamental for understanding load transfer mechanisms and for developing reliable analytical and numerical models for CFRP–steel-bonded joints. Second, the influence of corrosion damage on the effective bond length of CFRP–steel joints has not been fully clarified. In addition, the classification of corrosion damage adopted in existing studies is often insufficiently defined, making it difficult to directly correlate laboratory-induced corrosion with realistic corrosion severity levels encountered in service environments. Third, although interface modification techniques, such as introducing glass fibre sheets (GFS) into the adhesive layer, have shown potential for mitigating galvanic corrosion and enhancing bond performance, their effectiveness under different degrees of corrosion damage has not been systematically quantified.

To address the above knowledge gaps, this study presents an experimental investigation of the bond behaviour of CFRP–steel double-strap joints subjected to controlled corrosion damage. An accelerated electrochemical method, calibrated according to ISO 9223 [21] corrosivity categories, was employed to generate different levels of corrosion damage in CFRP–steel-bonded joints. Tensile tests were then conducted to evaluate the effects of corrosion degree, bond length, and the presence of GFS in the adhesive layer on failure modes, ultimate load capacity, and effective bond length. Digital image correlation (DIC) was used to obtain high-resolution strain distributions along the CFRP plates, enabling the establishment of a bond–slip model for corroded CFRP–steel interfaces. The results provide valuable insights into corrosion-induced bond degradation mechanisms and contribute to the development of more reliable design and durability assessment methods for CFRP-strengthened steel structures.

## 2. Experimental Program

### 2.1. Materials and Specimens

**Figure 1:**
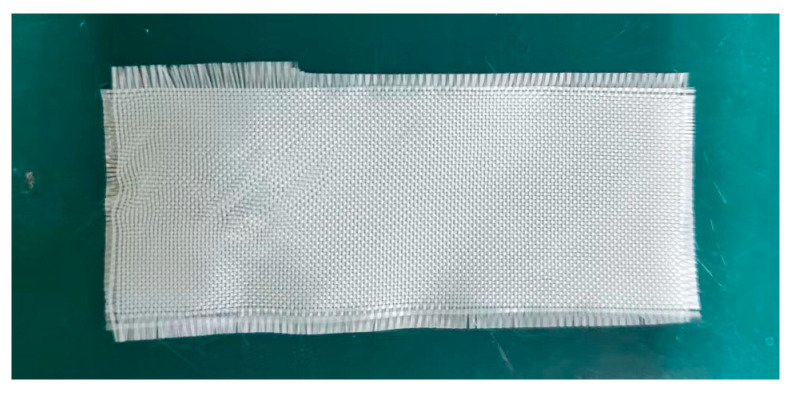
Glass fibre sheet (GFS) used in the testing.

All the materials used in this paper include hot-rolled Q345 steel (Tianjin Tuoyuze Technology Co., Ltd., Tianjin, China), CFRP plates (Lica-131, Nanjing Haituo Composite Materials Co., Ltd., Nanjing, China), glass fibre sheets (GFS) (0.1 mm thick woven fabric in orthotropic directions (Shanghai Huadong PTFE Products Factory, Shanghai, China), and a two-part epoxy adhesive (Araldite 420) manufactured by the Huntsman Corporation, The Woodlands, TX, USA. The CFRP plate, steel, and adhesive in this paper are the same as the ones used in the authors’ previous study [22], and the measured material properties according to ASTM D3039 [23], ASTM E8 [24], and ASTM D638-14 [25] are listed in Table 1. The glass fibres of GFS are woven in orthotropic directions, as shown in Figure 1, to assist in identifying GFS fragments in the failure sections shown in Section 3.3.

**Figure 2:**
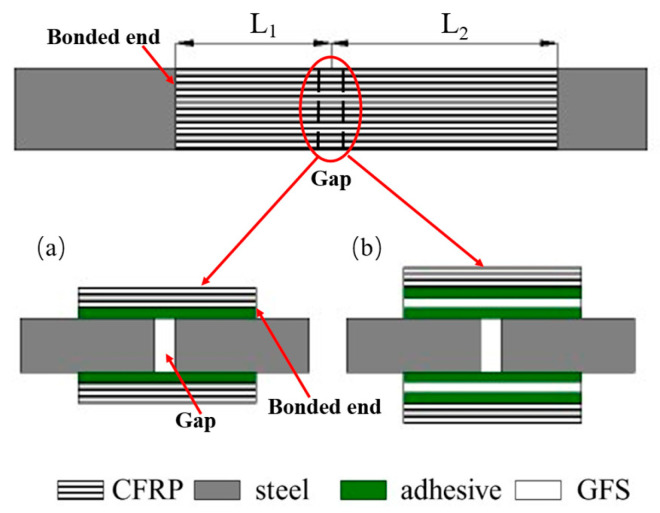
Schematic view of a typical double-strap joint specimen (**a**) without and (**b**) with GFS in the adhesive.

Two types of CFRP–steel double-strap joints with/without GFS in the adhesive are prepared, as shown in Figure 2, following a commonly adopted double-strap configuration to facilitate the investigation of interfacial bond behaviour [26]. All the steel plates are 30 mm wide and 15 mm thick. The width of the CFRP plate and the GFS are maintained in the same manner as the steel plate. The bond length (L_1_) of the CFRP plate for joints without GFS varies from 30 mm to 150 mm, while the bond length of the CFRP plate and GFS for joints with GFS remains 30 mm. The bond length (L_2_) of the other side, as shown in Figure 2, for both types of joints is 1.5 times that of L_1_ to ensure that the failure only occurred on one side of the joint (L_1_ side). The detailed procedure for preparing joints can be found in a previous study by the authors in [22]. The adhesive thickness of every specimen is measured with the method mentioned in [7] and listed in Section 2.3. When calculating the adhesive thickness of the joint with GFS in the adhesive, the thickness of the GFS shall be subtracted from the total thickness of the joint.

### 2.2. Specimen Grouping and Experimental Variables

To evaluate the effects of corrosion damage and GFS layer in the adhesive on the bond behaviour of CFRP–steel-bonded joints, 3 groups of experimental testing are performed in this study, as shown in Table 2. For Groups 1 and 2, all the specimens are designed with a bond length of 30 mm and exposed to 5 different degrees of corrosion damage. The method to generate corrosion damage through an accelerated procedure will be detailed in Section 2.3. The only difference between Group 1 and Group 2 is that specimens in Group 1 do not have GFS in the adhesive layer, while specimens in Group 2 do. By comparing Group 1 and 2, we can evaluate the effect of corrosion damage on the bond behaviour of CFRP–steel-bonded joints, either without or with GFS in the adhesive layer, respectively. In Group 3, the specimens’ bond length varies from 30 mm to 150 mm, and they are exposed to a very high degree (C5 according to ISO-9223 [21]) of corrosion damage. Group 3 intends to find out whether there exists an effective bond length for CFRP–steel joints under a severe corrosion level and to establish the corresponding bond–slip relationship for the joints.

For the specimen notations in Table 2, the first two letters describe whether GFS is present in the adhesive layer or not (CF = without GFS, and GF = with GFS). The following two digits represent the CFRP bond length. The two characters after the first hyphen represent the degree of corrosion damage according to ISO-9223 [21]. For the same joint, three identical specimens are tested for the purpose of repetition, which are identified by the last digit of the notation. For example, specimen GF30-CX-3 means the third repeating specimen of a joint with a bond length of 30 mm, and with GFS in the adhesive, exposing the degree of corrosion damage to CX. Specifically, specimens with a corrosion degree of C0 represent joints without corrosion damage, which serve as control specimens.

According to ISO-9223 [21], the corrosivity of a metal is divided into six categories, and the corresponding corrosion rates, *r*_corr_, are given in Table 3. In the present study, corrosion categories C2–C5 and CX were considered in the experimental program, while category C1 was not included, as its corrosion rate is extremely low and difficult to be reliably quantified under laboratory conditions within a reasonable testing duration. Considering that only the steel plates of the joint undergo corrosion, the mass loss of the joint is, therefore, equivalent to the mass loss of the steel plates. Thus, the maximum corrosion rate of each selected category in Table 3 was used to generate corrosivity of the corresponding testing groups in Table 2. Please note that the corrosion rate of category CX is set at *r*_corr_ = 3000 g/(m^2^·a) as a representative value within the CX range to facilitate accelerated testing, which is twice the maximum corrosion rate of category C5.

### 2.3. Accelerated Procedure to Generate Corrosion Damage

**Figure 3:**
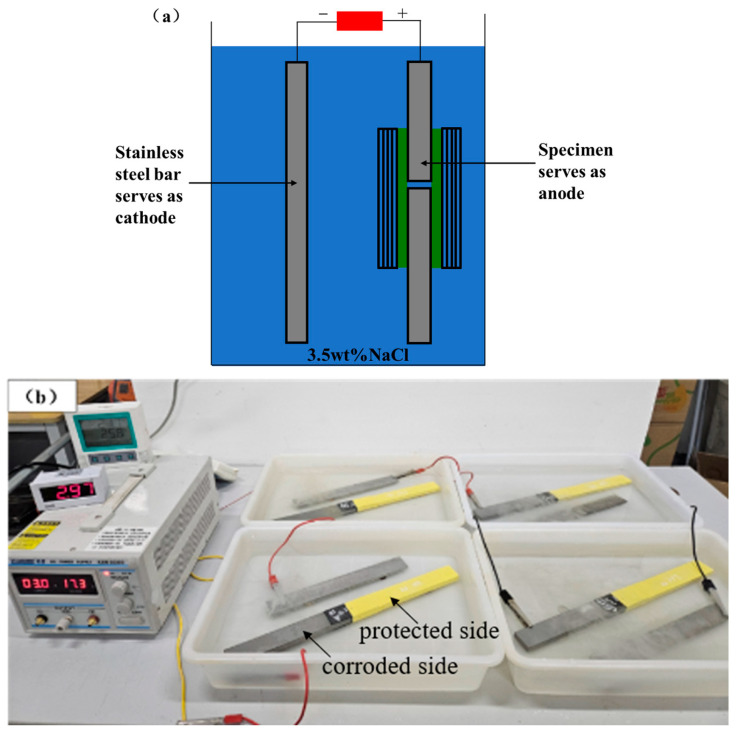
Setup for accelerated corrosion: (**a**) schematic representation, and (**b**) specimens connected in a series circuit.

The corrosion damage was generated by an accelerated electrochemical method, as shown in Figure 3. The setup includes a power supply, cathodes and anodes, an electrolyte solution, and plastic containers [27,28]. The CFRP–steel-bonded joint serves as the anode, undergoing an oxidation reaction, i.e., the anode generates electrons (Fe→Fe^2+^ + 2e^−^), whereas a stainless-steel bar is employed as the cathode that consumes electrons (O_2_ + 2H_2_O + 4e^−^ → 4OH^−^). A 3.5% sodium chloride solution is prepared as the electrolyte [29,30]. To drive the electric current from cathodes to anodes, an external constant current DC power supply was used (ZHAOXIN KXN-3020D, Shenzhen Zhaoxin Electronic Instruments and Equipment Co., Ltd., Shenzhen, China), with a maximum output voltage of 30 V and a maximum output current of 20 A). The power supply ensures a constant current throughout the entire test and can generate uniform corrosion damage to the bonded joint in a short time [27,31].

During the experiment, all joints were fully immersed in the electrolyte. To ensure that corrosion only occurs on the shorter bonded side of the joint, the shorter side was connected to the positive terminal of the power supply. The longer bonded side was wrapped and protected by insulating tape, as shown in Figure 3b. A constant current of 3 A was set throughout the whole test, and serial connections were used between repeated specimens to ensure a current of 3 A passed through all the specimens. It was observed that the electric current remained stable throughout all the experiments. When the predetermined electrochemical processing time was reached, the power supply was automatically switched off, and all specimens were subsequently cleaned and weighed. The exposure durations reported in Table 2 were calculated based on the governing equations, and the timing error associated with specimen removal was negligible compared with the overall exposure period.

The mass loss of the joint can be predicted based on the exposed area of the steel, and the results are shown in the “Predicted corrosion mass loss” column in Table 4. According to Faraday’s law in Equation (1), given an electric current of 3 A, the time duration required to achieve a specific steel plate mass loss can be determined. The calculated results are shown in the “Exposure duration” column in Table 2. Please note that the mass loss of the steel plate under category C1 is less than 1 g; it cannot be accurately measured in the laboratory, and therefore, no measurements were conducted for this degree of corrosion damage:

(1) Δm=MIt/zF

where Δm is the steel mass loss in g; M is the atomic weight in g/mol, which is 55.9 g/mol for steel; I is the input current, which is 3 A in test; t is the exposure duration in s; z is the number of electrons for the iron ion, which is 2 for Fe^2+^; and F is Faraday’s constant, which is 96,500 C/mol.

Taking Specimen CF30-C4 as an example to illustrate the calculation process, the exposed steel area of this specimen is 0.01485 m^2^, and the corrosion rate for C4 is set at 650 g/(m^2^·a) in this study. Therefore, the predicted corrosion mass is calculated by multiplying the exposed area by the corrosion rate, resulting in a value of Δm = 9.6525 g. To achieve this corrosion mass, the exposure duration can be determined using Equation (1), with *M* = 55.9, *I* = 3 A, *z* = 2, and *F* = 96,500 C/mol, resulting in a value of 11,108.72 s, as shown in the “Calculated Exposure duration” column in Table 2.

### 2.4. Instrumentation and Loading Procedure

**Figure 4:**
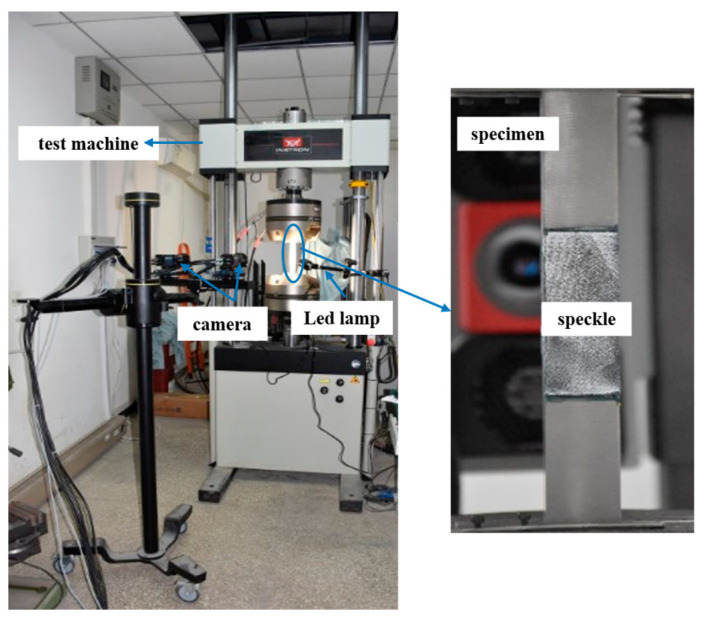
Test setup for tensile loading of CFRP–steel joint specimens.

All specimens after corrosion were tested in tension using an Instron 8802 hydraulic testing machine (INSTRON, Norwood, MA, USA) by displacement control at a loading rate of 1 mm/min. The test was continued until the failure of the specimen. Digital image correlation (VIC-3D M8.9, Correlated Solutions Inc., Columbia, SC, USA) was used to track the full-field surface displacement and strain of the CFRP plate of the specimens. The tensile loading test setup is shown in Figure 4.

## 3. Results and Discussion

Test results are presented in Table 4, including the measured adhesive thickness, measured steel mass loss, ultimate load, and failure modes of the joint specimens. The load reduction (%) was calculated based on the average ultimate load of three repeated specimens. The measurement accuracies of mass, adhesive thickness, and ultimate load were 0.01 g, 0.01 mm, and 0.01 kN, respectively.

### 3.1. Corrosion Morphology and Mass Loss

**Figure 5:**
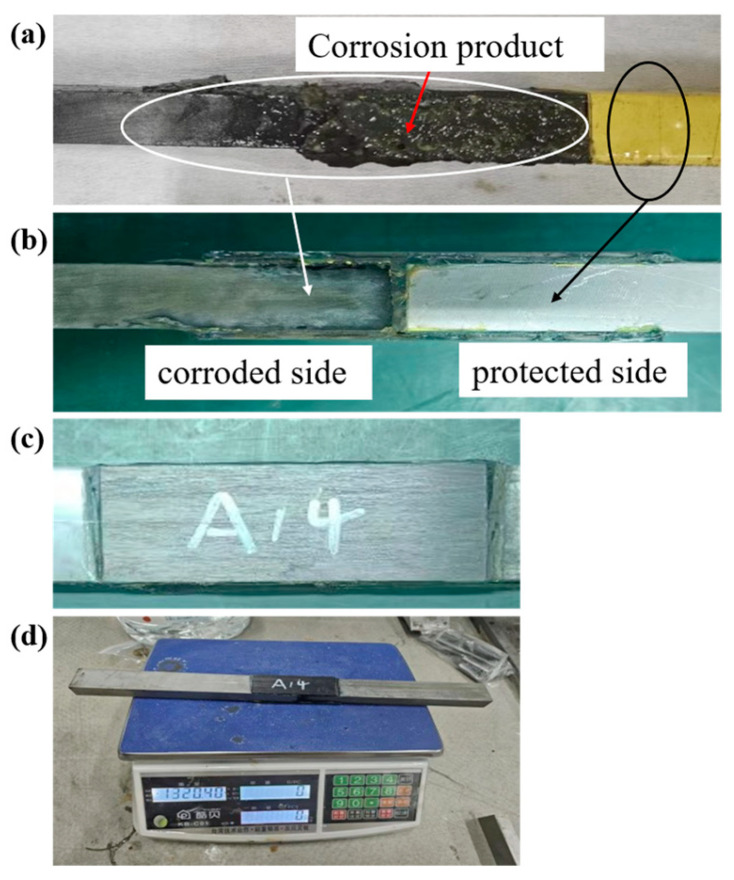
After an accelerated corrosion test (**a**) with the corrosion product attached to the shorter bonded side of the joint (**b**); obvious corrosion on the shorter bonded side and no evident change occurred on the protected side (**c**); no change in CFRP plate (**d**); the joint was weighed after cleaning.

A typical corrosion morphology of a joint is shown in Figure 5. Before cleaning, it can be clearly seen that the corrosion product is attached to the steel surface of the shorter bonded side of the joint (as shown in Figure 5a). After cleaning the corrosion product and removing the protected tape, the steel surfaces are shown in Figure 5b. It shows that the steel of the shorter bonded side (unprotected side) is corroded, and the corrosion pits can be found on the steel. The steel of the longer bonded side (protected side) shows no evident change, which indicates that the steel of the longer bonded side is not corroded. As the CFRP plate has excellent corrosion resistance, compared to steel, no obvious change is observed (as shown in Figure 5c), even if the CFRP plate is exposed to the corrosion environment. After cleaning and drying, the joint is weighed and the mass is recorded to calculate corrosion mass loss.

**Figure 6:**
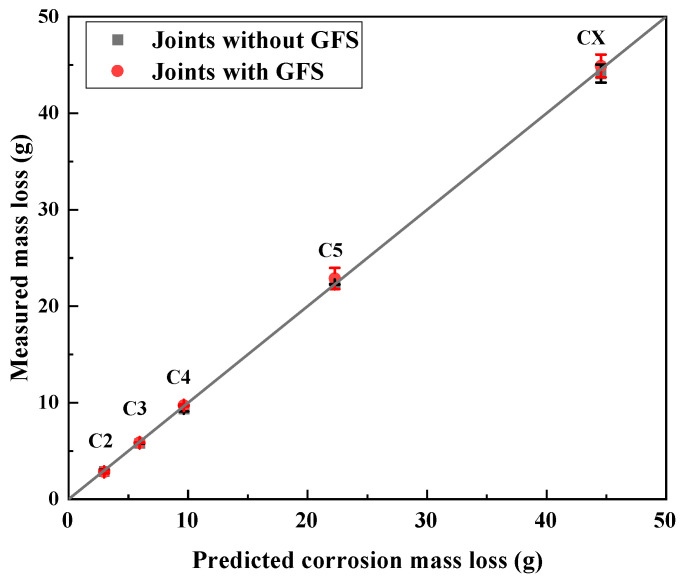
Measured vs. predicted corrosion mass loss of joints under different corrosivity categories (joints with and without GFS in adhesive layer, i.e., Groups 1 and 2 specimens).

**Figure 7:**
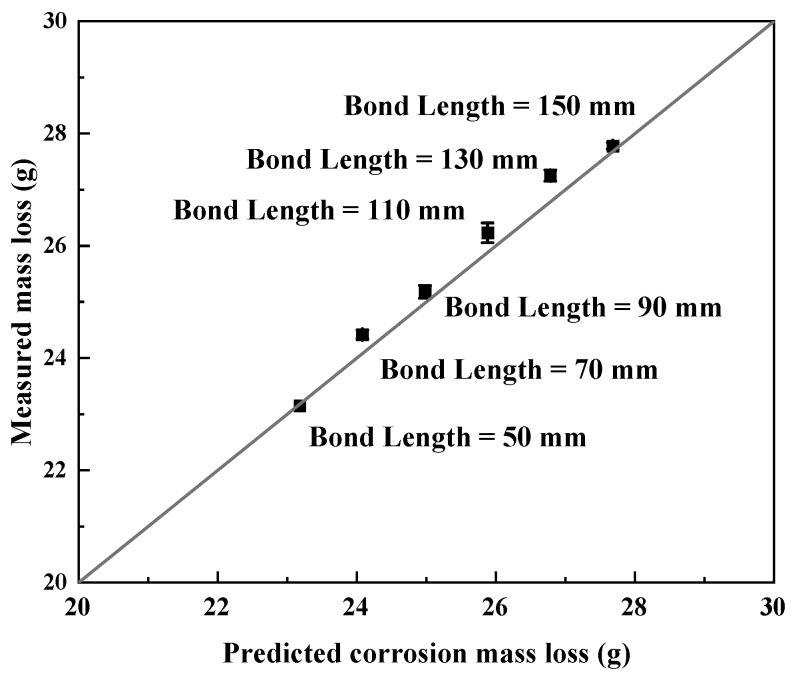
Measured vs. predicted corrosion mass loss of joints with different bond lengths (joints without GFS in adhesive layer under category CX, i.e., Group 3 specimens).

The comparison between predicted and measured corrosion mass loss under different corrosion categories but with the same bond length (Groups 1 and 2 specimens) is shown in Figure 6. The comparison between predicted and measured corrosion mass loss under the same category of CX but with different bond lengths (Group 3 specimens) is presented in Figure 7. The measured corrosion mass loss of all specimens agrees well with the predicted values, which indicates that the electrochemical method used in this research is reliable. The close agreement between the measured and predicted corrosion mass loss observed in Figure 6 and Figure 7 provides further insight into the corrosion mechanism of the CFRP–steel-bonded joints. The consistency across different corrosion categories, bond lengths, and the presence or absence of GFS suggests that the corrosion mass loss is predominantly governed by the electrochemical parameters and the exposed steel surface, rather than by the CFRP bond length or the incorporation of GFS in the adhesive layer. These observations also support the assumption adopted in this study that corrosion-induced mass loss is dominated by the steel component of the joint.

### 3.2. Effect of Corrosion Damage

**Figure 8:**
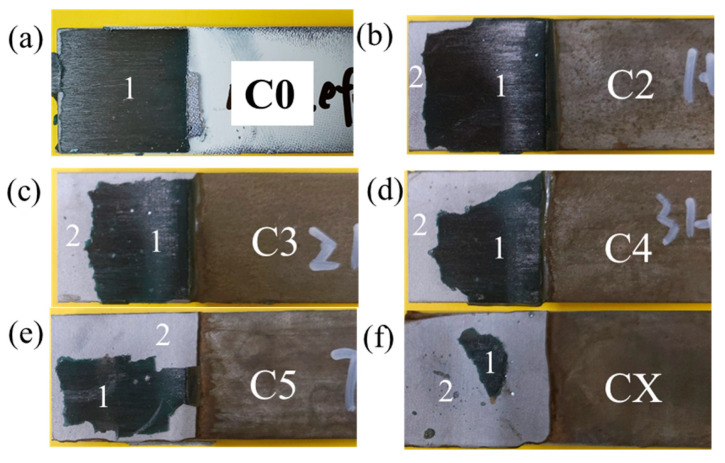
Failure modes of CFRP–steel-bonded joints without GFS in the adhesive layer under a corrosion damage of (**a**) C0 (no corrosion), (**b**) category C2, (**c**) category C3, (**d**) category C4, (**e**) category C5, and (**f**) category CX (Notes: 1 indicates steel–adhesive interface debonding and 2 indicates CFRP delamination).

The specimens in Group 1 are used for the discussions in this section. For the specimen without corrosion damage, as shown in Figure 8a, the failure mode is CFRP delamination. The failure modes of the corroded joints are a mixture of CFRP delamination and steel–adhesive interface debonding, as shown in Figure 8b–f. It can also be found in Figure 8 that, with the corrosion damage becoming worse (from category C2 to CX), the area of steel–adhesive interface debonding increases. Therefore, it can be concluded that when corroded, CFRP–steel-bonded joints tend to fail with steel–adhesive interface debonding, due to the interface weakening by the corrosive environment [32,33].

**Figure 9:**
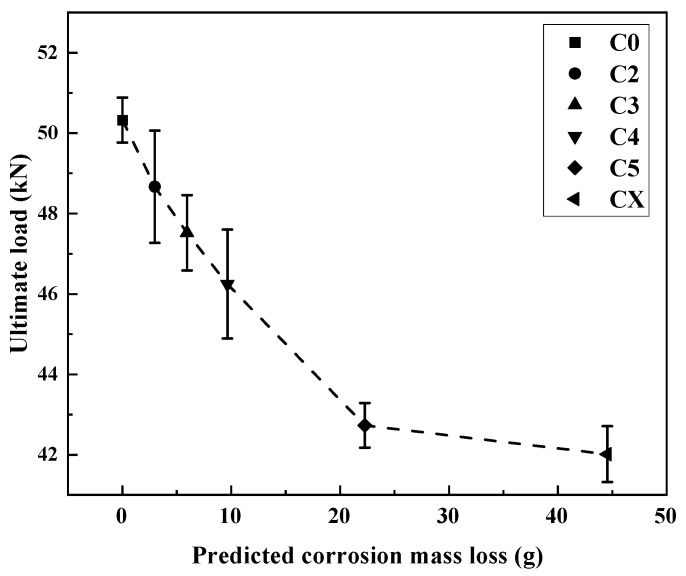
Ultimate loads vs. corrosion damage degree for CFRP–steel-bonded joints.

The ultimate loads of the CFRP–steel-bonded joints with various degrees of corrosion damage are shown in Figure 9. Generally, the ultimate load decreases as the corrosion increases. Specifically, the ultimate load decreases from 50.32 kN for a non-corroded joint to 42.01 kN for a joint with the maximum corrosion damage of category CX, showing a load reduction of 16.51%. It is also observed that the load reduction becomes slower as the degree of corrosion damage increases. For example, the load reduction is about 11.8% when the damage increases from C2 to C5, with a mass loss increase from 2.97 g (C2) to 22.28 g (C5). On the other hand, the load reduction is just 1.43% from C5 to CX, with almost doubled mass loss.

The evolution of failure modes shown in Figure 8 reflects a progressive degradation of the steel–adhesive interface with increasing corrosion damage. For the non-corroded joint (C0), failure occurs predominantly by CFRP delamination, indicating that the interfacial bond strength exceeds the interlaminar strength of the CFRP laminate. As corrosion damage increases (C2 to C5), mixed failure modes consisting of CFRP delamination and steel–adhesive interface debonding are observed, suggesting a gradual weakening of the interface due to corrosion-induced deterioration. Under the most severe corrosion condition (CX), the failure mode is dominated by steel–adhesive interface debonding, with a significantly enlarged debonded area. This indicates that the steel–adhesive interface becomes the critical weak link governing joint failure.

The corresponding trend in ultimate load shown in Figure 9 is consistent with this failure mode transition. While the ultimate load decreases markedly at lower-to-moderate corrosion levels, the rate of load reduction diminishes as corrosion damage further increases. This behaviour can be attributed to the shift in the governing failure mechanism. Once the steel–adhesive interface controls the failure process, further corrosion primarily enlarges the debonded area rather than causing a proportional reduction in load-carrying capacity. As a result, the ultimate load becomes less sensitive to additional corrosion damage at high degradation levels.

### 3.3. Effect of GFS in the Adhesive Layer

**Figure 10:**
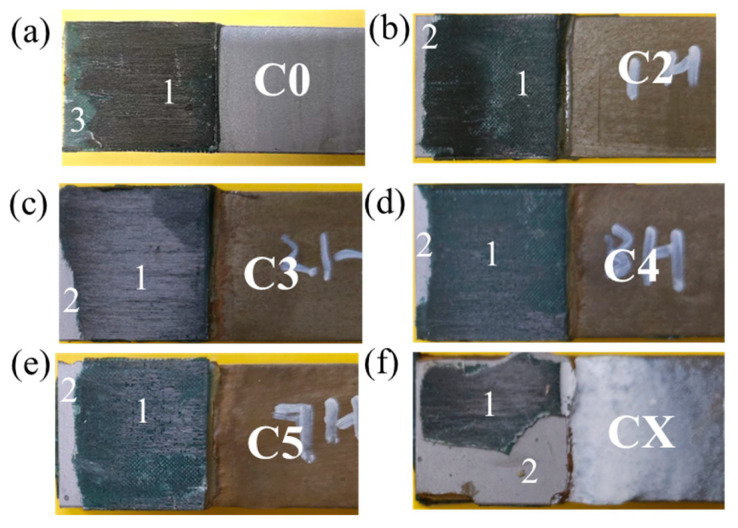
Failure modes of CFRP–steel-bonded joints with GFS in the adhesive layer under a corrosion damage of (**a**) C0 (no corrosion), (**b**) category C2, (**c**) category C3, (**d**) category C4, (**e**) category C5, and (**f**) category CX (Notes: 1 indicates steel–adhesive interface debonding, 2 indicates CFRP delamination, and 3 indicates GFS fragments).

The specimens in Group 2 are used for the discussions in this section. For uncorroded joints with GFS in the adhesive layer, the failure mode is CFRP delamination with GFS fragments observed on the fracture surface, as shown in Figure 10a. All the corroded specimens with GFS in the adhesive layer exhibit a mixture failure mode of CFRP delamination and steel–adhesive interface debonding, as shown in Figure 10b–f. For corrosivity category C2 to C5, the failure mode is dominated by CFRP delamination, while only a small area of steel–adhesive debonding can be observed on the fracture damage. For category CX, although the area of steel–adhesive interface debonding increases, the failure is still dominated by CFRP delamination, as shown in Figure 10f. Comparing the failure modes of CFRP–steel-bonded joints without GFS (as shown in Figure 8) and those with GFS in the adhesive layer (as shown in Figure 10), the area of steel–adhesive interface debonding of joints with GFS is always smaller than that of joints without GFS. This phenomenon indicates that GFS in the adhesive layer could enhance the bonding between the steel and adhesive to a certain extent, thereby mitigating corrosion-induced interfacial degradation and delaying the development of steel–adhesive interface debonding. This enhancement can be attributed to the presence of GFS within the adhesive layer, which improves stress redistribution and crack-bridging capability at the steel–adhesive interface. As a result, the propagation of interfacial debonding is restrained, even under corrosion exposure, and failure tends to be governed by CFRP delamination.

**Figure 11:**
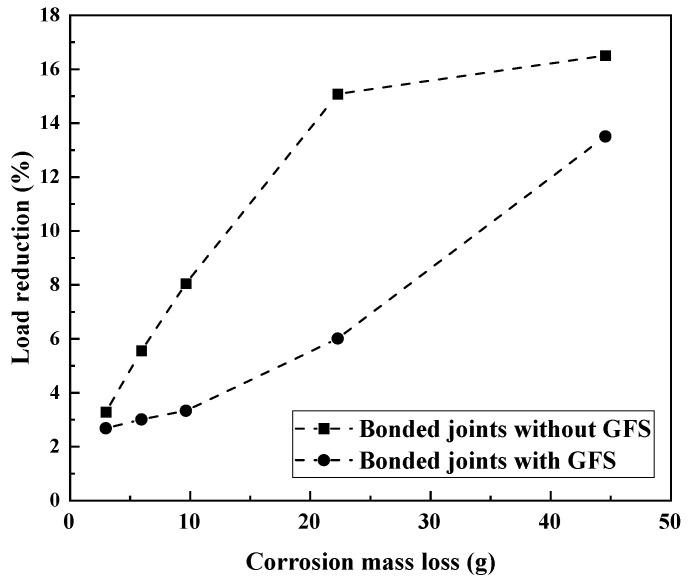
Load reduction vs. corrosion damage degree for CFRP–steel-bonded joints with and without GFS in the adhesive layer.

The ultimate load reduction in corroded GF and CF specimens compared to the non-corroded ones under various degrees of corrosion damage is shown in Figure 11. It is obvious that, for both joints with and without GFS, the ultimate load reduction increases when the corrosion damage becomes severe. However, the load reduction rate presents a different pattern, i.e., for joints without GFS, the reduction rate slows down with corrosion damage increases, while for joints with GFS, the reduction rate accelerates. This trend is related to the failure modes. For joints with GFS, the failure is dominated by CFRP delamination when subjected to the corrosion damage of C2~C5, and thus, the change in load reduction is relatively small. When the joint is subjected to more severe corrosion damage of CX, the steel–adhesive interface debonding obviously enlarges, and thus, the load reduction increases significantly. This indicates that, once corrosion-induced interfacial degradation becomes sufficiently severe, the beneficial effect of GFS is partially diminished, and the steel–adhesive interface begins to play a more dominant role in governing joint failure. Another observation of Figure 11 is that, when undergoing the same degree of corrosion damage, the joints with GFS always perform better with less load reduction than the joints without GFS. Similar results were also reported in the literature [31].

### 3.4. Effect of CFRP Bond Length

**Figure 12:**
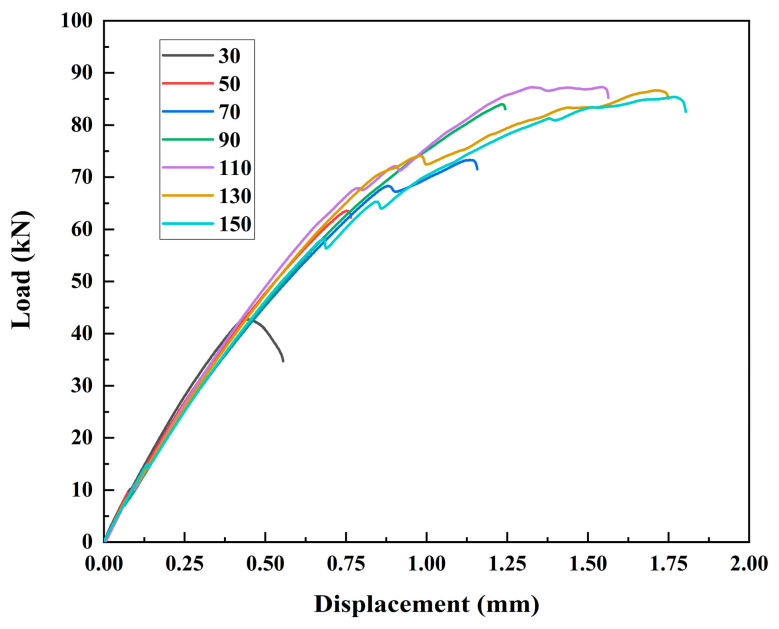
Load–displacement curves for CFRP–steel-bonded joints with various bond lengths under a corrosion damage of C5.

The specimens in Group 3 are used for the discussions in this section. These specimens have no GFS and are subjected to the same corrosion damage of C5. The typical load–displacement curves of specimens with different bond lengths are shown in Figure 12. The only difference among all the load–displacement curves is whether the loading curve has a plateau stage before joint failure. For corroded joints with bond lengths from 30 mm to 90 mm, the specimens fail in a brittle way. The load first increases with the displacement and then drops immediately after the load reaches its peak. The displacement at failure increases with the bond length. For example, specimen CF90-C5-1 fails with a displacement of 1.25 mm, which is 2.3 times that of specimen CF30-C5-1 (0.55 mm). For corroded joints with greater bond lengths from 110 mm to 150 mm, the load first grows and then reaches a plateau after a certain displacement. The appearance of a plateau stage in the load–displacement response indicates a transition from brittle failure to a progressive failure process, which is associated with gradual interfacial debonding and slip development along the bonded length. This behaviour suggests that the interface no longer fails instantaneously, but instead undergoes a more distributed stress transfer process prior to ultimate failure. Their displacement at failure gradually becomes stable with the increase in bond length. For example, specimens CF130-C5-1 and CF150-C5-1 fail with a similar displacement of 1.75 mm and 1.80 mm, respectively.

In this study, the effective bond length is defined as the bond length beyond which further increase in bond length does not lead to noticeable changes in either the ultimate load or the failure displacement. From Table 4’s ultimate load and averaged ultimate load column, the ultimate load of the specimens in Group 3 remains almost stable when the bond length exceeds 110 mm, which indicates there is an effective bond length of these corroded CFRP–steel-bonded joints. Combined with the load–displacement curve in Figure 12, load–displacement curves for joints with bond length 130 mm and 150 mm have a similar trend and failure displacement. Altogether, it can be concluded that the effective bond length for CFRP–steel joints with category C5 corrosion damage is no less than 130 mm. The corresponding ultimate load of the corroded joint is 86.10 kN. The effective bond length and corresponding ultimate load of the non-corroded joint with the same material and CFRP bonded width, calculated with the theoretical model from previous research [34], are 126.59 mm and 124.84 kN. After corrosion degradation, the effective bond length of CFRP–steel-bonded joints increases, but the ultimate load decreases. As corrosion damage leads to a degradation in bonding performance, a longer bond length is required to complete the transfer process of interface stress and achieve a corresponding bearing capacity. Therefore, the effective bond length of corrosion-damaged joints is slightly larger than that of non-corroded joints. As the performance of adhesive and the adhesive–steel bond is weakened by corrosion damage, the ultimate load of corroded joints decreases, even with a longer effective bond length.

**Figure 13:**
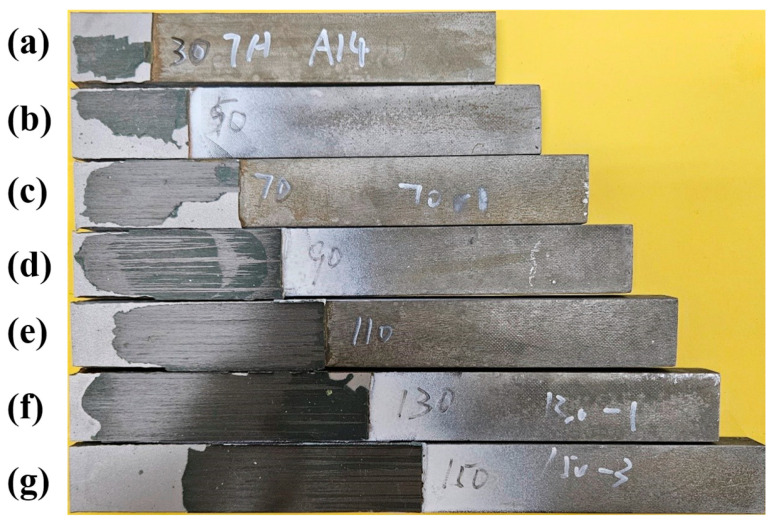
Failure modes of corroded (corrosive category C5) CFRP–steel-bonded joints with various bond lengths of (**a**) 30 mm, (**b**) 50 mm, (**c**) 70 mm, (**d**) 90 mm, (**e**) 110 mm, (**f**) 130 mm, and (**g**) 150 mm.

All the corroded joints fail with a mixture of CFRP delamination and steel–adhesive interface debonding, as shown in Figure 13. This indicates that, once corrosion-induced degradation governs the interfacial behaviour, variations in bond length mainly influence the deformation and load redistribution process, rather than altering the dominant failure mode of the joint. This is because the penetration of water molecules from the corrosive environment will weaken the adhesive [31,32], promoting the steel–adhesive interface debonding.

The degradation of the adhesive and the steel–adhesive bond observed in this study can be attributed to several corrosion-related mechanisms. Exposure to a corrosive environment facilitates the ingress of moisture and corrosive agents into the adhesive layer, primarily from the exposed edges of the bonded joint and potentially through microscopic defects within the adhesive. This ingress may lead to adhesive plasticization and a reduction in stiffness and strength, as reported in previous studies [28,31,32].

In addition, corrosion of the steel substrate can generate corrosion products at the steel–adhesive interface, causing local stress concentrations and disrupting interfacial adhesion, which further promotes the penetration of corrosive agents along the interface. These coupled effects weaken the load transfer capability of the interface and promote the transition from CFRP delamination to steel–adhesive interface debonding with increasing corrosion damage. The experimentally observed reductions in ultimate load and increase in effective bond length are consistent with these mechanisms, indicating that corrosion primarily degrades the mechanical performance of the adhesive layer and the steel–adhesive bond rather than altering the CFRP material itself.

## 4. Bond–Slip Behaviour

Bond–slip behaviour refers to the relationship between the interface shear stress along the bondline and its corresponding slip of the bonded joint. It provides insight into how the load is transferred between CFRP and steel and the associated failure mechanism of bonded joints. The bond–slip model for corroded CFRP–steel-bonded joints is established in this section.

### 4.1. Longitudinal Axial Strain Along the CFRP Plate

As discussed in Section 3.4, the load–displacement curves of corroded joints with various bond lengths can be divided into two types, i.e., with and without a plateau loading stage. Thus, the following discussions on the longitudinal axial strain contours along the CFRP plate are also presented separately.

**Figure 14:**
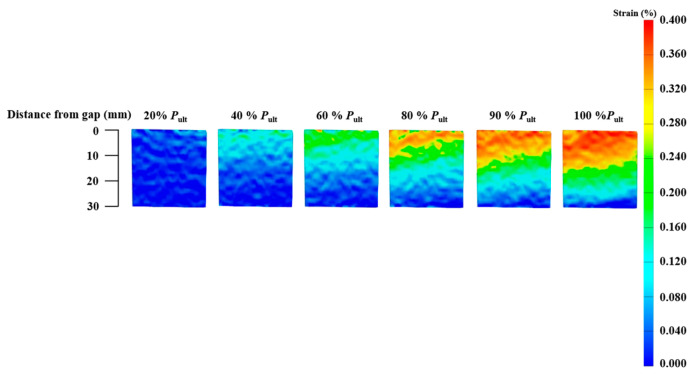
CFRP axial strain contours at various stages of loading (Specimen CF30-C5-1).

**Figure 15:**
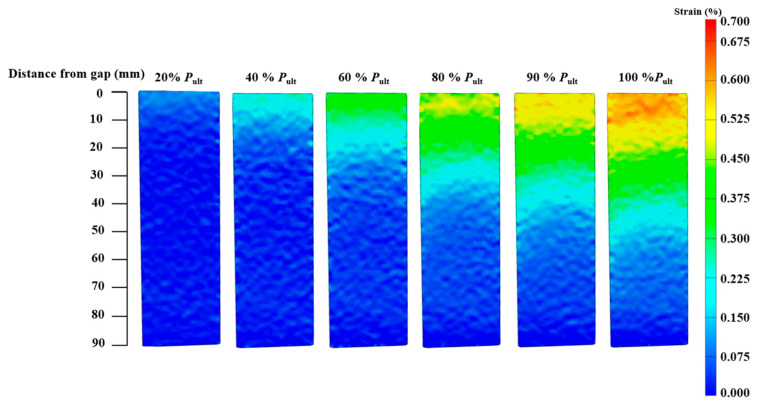
CFRP axial strain contours at various stages of loading (Specimen CF90-C5-1).

**Figure 16:**
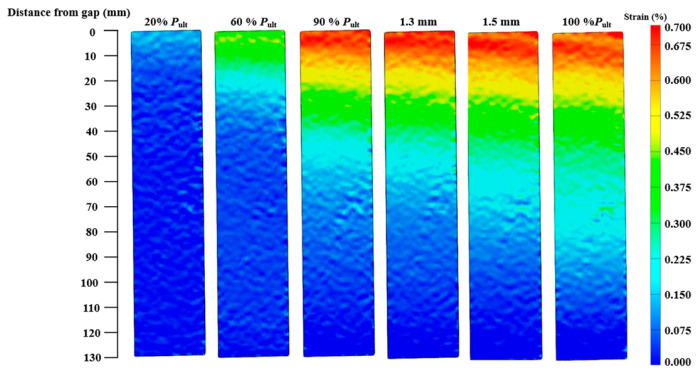
CFRP axial strain contours at various stages of loading (Specimen CF130-C5-1).

**Figure 17:**
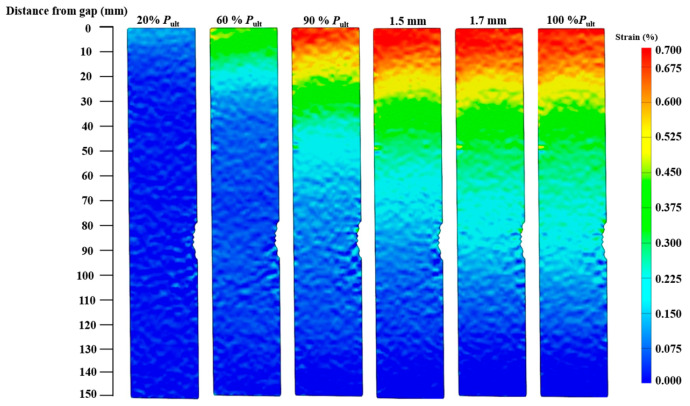
CFRP axial strain contours at various stages of loading (Specimen CF150-C5-1).

For joints without a plateau loading stage, the strain contours of specimens CF-C5-30-1 and CF-C5-90-1 are presented in Figure 14 and Figure 15, respectively. The load level in the Figure 14, Figure 15, Figure 16 and Figure 17 is defined as the ratio of applied load to the ultimate load capacity of the specimen. The CFRP strain contours of these two specimens show a similar pattern. At a lower load level, the CFRP strain is relatively small over the entire CFRP plate. With the increase in the load level, the strain at the joint gap increases. It can also be observed that the range of high strain expands along the bond length from the gap to the bonded end as the load level increases.

For joints with a plateau loading stage, the strain contours of specimens CF130-C5-1 and CF150-C5-1 are presented in Figure 16 and Figure 17, respectively. When the joint is within the linear stage (before 90% ultimate load of the specimen), the CFRP strain at the joint gap increases as the load level goes up, and the range of high strain expands along the bond length from the gap to the bonded end. After reaching the plateau loading stage, the maximum strain remains stable while the range of the high strain still expands from the joint gap to the bonded end.

**Figure 18:**
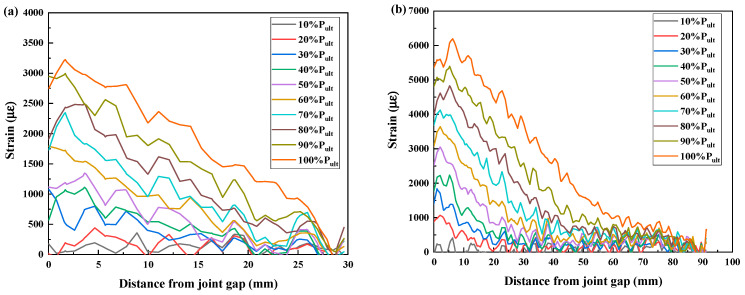

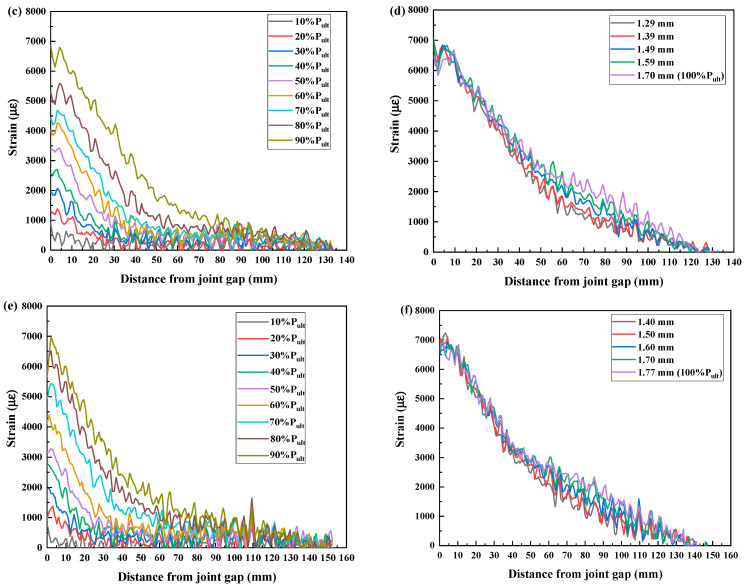
CFRP axial strain distributions of corroded specimens: (**a**) Specimen CF30-C5-1, (**b**) Specimen CF90-C5-1, (**c**) Specimen CF130-C5-1, linear stage, (**d**) Specimen CF130-C5-1, plateau stage, (**e**) Specimen CF150-C5-1, linear stage, and (**f**) Specimen CF150-C5-1, plateau stage.

CFRP strain distributions under different load levels of specimens CF30-C5-1, CF50-C5-1, CF130-C5-1, and CF150-C5-1 are extracted from strain contours and plotted in Figure 18. It can be found in Figure 18a,b that, at the same load level, the maximum CFRP strain occurs at the joint gap, and the strain decreases towards the bonded end. With the increase in the load level, the maximum CFRP strain also rises. When the bond length increases from 30 mm to 90 mm, the maximum CFRP strain also increases from 3226.07 με to 6196.63 με. This is reasonable because a longer bond length yields a greater ultimate load of joints, which leads to higher strain at the joint gap. For specimens with a bond length longer than 90 mm, as discussed before, the CFRP strains of the joint at both linear and plateau loading stages of the load–displacement curve were recorded. For the linear stage, as shown in Figure 18c,e, the CFRP strains have a similar pattern as those for joints with a shorter bond length. For the plateau loading stage, as shown in Figure 18d,f, as the load remains stable, the maximum CFRP strain remains constant with the increase in joint displacement, while the high strain area expands towards the bonded end. For example, when the bond length increases from 130 mm to 150 mm, the maximum CFRP strain is almost the same, i.e., 6967.21 με for specimen CF130-C5-1 and 6903.43 με for specimen CF150-C5-1.

Please note that the strains on the CFRP plates were obtained by the 3D-DIC method, which gives a high resolution, i.e., the minimum measurable distance between two points on the CFRP plate can be down to 1 mm. However, the strain distributions in Figure 18 exhibit some fluctuations, which need to be properly smoothed before being used for bond–slip model development [35,36]. Moreover, the same maximum CFRP strain and similar strain distribution for specimen CF130-C5 and CF150-C5 indicate that 130 mm has exceeded the effective bond length of corroded CFRP–steel-bonded joints.

### 4.2. Establishment of the Bond–Slip Model

**Figure 19:**
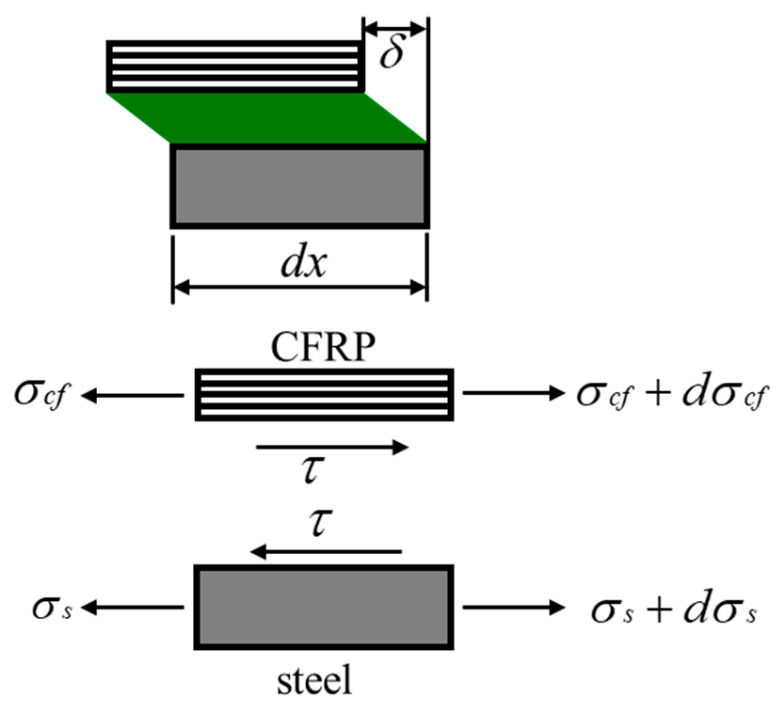
Deformation and equilibrium conditions of an infinitesimal element of the joint.

The determination of the local shear stress at the CFRP–steel interface and the associated relative slip is the key to obtaining the bond–slip model. The deformation and equilibrium conditions of an infinitesimal element of the joint are shown in Figure 19. The interfacial shear stress and the slip can be estimated from the CFRP axial strains using Equations (2)–(4) [7,31].

(2) $$\tau =E_{\mathrm{cf}}t_{\mathrm{cf}}\frac{d\varepsilon }{dx}$$

(3) $$\tau_{i}=E_{\mathrm{cf}}t_{\mathrm{cf}}\frac{\varepsilon_{i+1}-\varepsilon_{i}}{\Delta x}$$

(4) $$\delta_{i}=\delta_{i-1}+\frac{\varepsilon_{i+1}+\varepsilon_{i}}{2}\Delta x$$

where *E*_cf_ and *t*_cf_ are CFRP elastic modulus and thickness, $\varepsilon_{i-1}$ and $\varepsilon_{i}$ are CFRP strains, i=1,2,3,…, and Δx is the distance between measurement points. The average slip $\delta_{i}$ is calculated as the incremental sum of the CFRP extension and $\delta_{0}=0$.

**Figure 20:**
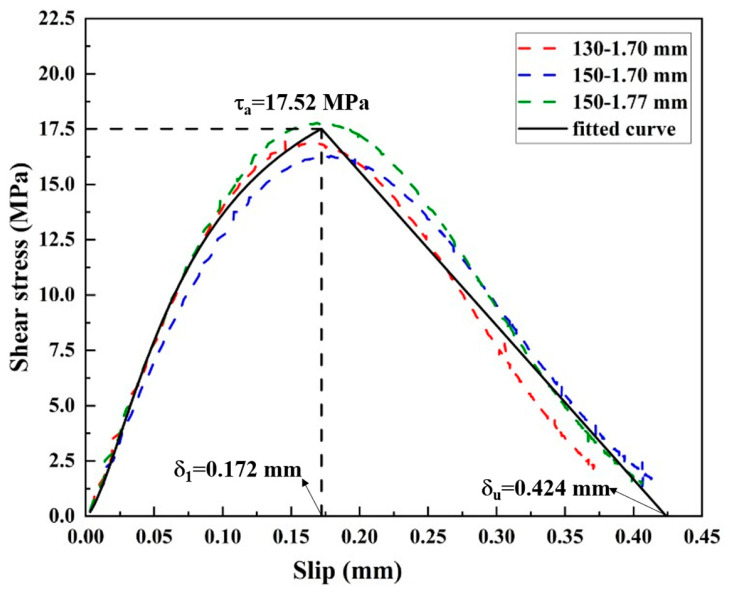
Shear stress–slip curves for corroded CFRP–steel joint experimental and fitted model.

Figure 20 shows the interfacial bond–slip relationships obtained from specimens CF130-C5-1 and CF150-C5-1 under the plateau loading stage. The overall development trend of the bond–slip relationship is clear: the shear stress rises gradually with the increase in relative slip in the initial stage until the peak shear stress is reached. The peak shear stress is around 15 MPa to 17 MPa, and the corresponding slip is around 0.17 mm. Thereafter, as the slip of the interface continues to increase, the interfacial shear stress decreases gradually to zero. Even from different loading conditions and specimens, it can be seen that the bond–slip curves are slightly different from each other. This is because both specimens CF130-C5-1 and CF150-C5-1 have a bond length longer than the effective bond length, and the shear transfer process is completed within the tested bond length [7,36].

The fitted model is also shown in Figure 20, and the fitting is subjected to the principles as reported in [36]. It should be noted that the bond–slip relationship developed herein is derived based on the experimental results of Group 3 specimens, which do not incorporate GFS in the adhesive layer, and is therefore applicable to corroded CFRP–steel-bonded joints without GFS. Although previous studies have shown that corrosion of steel surfaces is often spatially non-uniform [16,17], existing bond–slip modelling approaches commonly adopt an equivalent interfacial response derived from global test results, which is also followed in the present study. The data in the ascending and descending branches of the bond–slip curves are fitted with an exponential curve, as shown in Equation (5), and a linear curve, as shown in Equation (6), respectively. $\tau_{a}$ is the maximum shear stress, which is 17.52 MPa. $\delta_{1}$ is the slip at damage initiation corresponding to the maximum shear stress, which is 0.172 mm; $\delta_{u}$ is the slip limit, which is 0.424 mm. The fracture energy *G*_f_ is the area surrounded by the bond–slip curve, which is calculated as 5.49 N/mm:

(5) $$\tau^{-1}=0.044+\frac{9.15e^{-4}}{\delta^{1.5}}$$

(6) $$\tau =29.49-69.55\delta \;\;\delta_{1}\leq \delta \leq \delta_{u}$$

Compared to the bond–slip model of non-corroded joints presented in the previous study [34], there are three main changes for the bond–slip model of joints with corrosion damage. Firstly, the peak shear stress shows a significant drop due to environmental degradation. For example, the maximum interface shear stress is 17.52 MPa, only 65% of that of a non-corroded joint. Secondly, it is noted that the bond–slip response exhibits a gradual descending stage after the shear stress reaches its peak, which means the failure is progressive and has a softening stage, while the shear stress drops to zero immediately after the peak for non-corroded joints. Finally, the fracture energy also shows a deterioration. In the absence of corrosion-induced damage, the fracture energy of bonded joints can be considered identical for joints fabricated with the same materials. Moreover, the fracture energy is linearly proportional to the adhesive thickness [37]. Therefore, based on the results reported in [34], and accounting for the difference in adhesive layer thickness, the fracture energy of the bonded joints investigated in the present study under non-corroded conditions can be inferred to be 10.29 N/mm. After corrosion exposure, the fracture energy obtained in this study decreases to 5.49 N/mm. All these features of the obtained bond–slip relationship imply that the corrosion damage is harmful to the adhesive and the bond interface, resulting in the degradation of the bond performance. These changes in the bond–slip response can be attributed to corrosion-induced degradation of the adhesive layer and the steel–adhesive interface, which reduces the interfacial shear transfer capacity and promotes progressive debonding. As a result, the load transfer mechanism becomes more distributed along the bonded length, leading to a softened post-peak response and reduced fracture energy compared to non-corroded joints.

## 5. Conclusions

This paper has presented a study on the bond behaviour of CFRP–steel-bonded joints with corrosion damage. The joints were first subjected to corrosion through an accelerated electrochemical process. The corrosion damage level was controlled according to the international standard. The corroded joints were then tested in tension to investigate the bond behaviours, including the failure modes, ultimate load, effective bond length, and bond–slip behaviour. Furthermore, GFS was introduced into the adhesive layer, and its effect on the bond behaviour was also investigated. Based on the observations and the results of the experimental program, the following conclusions can be drawn:

1. The electrochemical corrosion method adopted in this study enables the controlled and efficient generation of different corrosion damage levels calibrated to ISO 9223 [21], providing a practical basis for investigating corrosion-induced degradation of CFRP–steel-bonded joints. The measured steel plate mass loss is almost consistent with the predicted mass loss. It is observed that the obvious corrosion only occurs on the steel plates, and there are corrosion pits on the steel surfaces. The morphology of the CFRP plate does not change significantly.
2. As the degree of corrosion damage increases, the ultimate load of CFRP–steel-bonded joints without GFS with the same bond length (30 mm) declines up to 16.51%, but the reduction rate slows down. The failure mode changes from CFRP delamination to steel–adhesive interface debonding with an increase in damage degree.
3. The failure mode of joints with GFS is CFRP delamination for all corrosion damage categories except for CX, which exhibits steel–adhesive interface debonding. GFS in the adhesive layer can enhance the bond performance of the joints under corrosion conditions. Given a specific corrosion damage and bond length, the ultimate load of joints with GFS is greater than that of joints without GFS, with an increase of 8.26% for a very high corrosion damage level (C5).
4. For joints with a very high level of corrosion damage (category C5), the ultimate load of the joints without GFS first increases and then remains constant (around 86 kN) as the bond length increases. The effective bond length of the corroded joint is about 130 mm, which is a little larger than the non-corroded joint. The failure mode is a mixture of CFRP delamination and steel–adhesive interface debonding, which is independent of the bond length.
5. A bond–slip model is established for corroded CFRP–steel-bonded joints. The relationship is greatly affected by the corrosion damage, which has a much lower maximum interfacial shear stress (17.52 MPa), a smaller fracture energy (5.49 N/mm), and a progressive softening failing process.

Based on the experimental findings, effective corrosion mitigation for CFRP–steel-bonded joints should focus not only on reducing corrosion of the steel substrate, but also on preserving the mechanical integrity of the steel–adhesive interface, as corrosion-induced interfacial degradation was found to govern failure behaviour and load-carrying capacity reduction. Further investigation is needed to assess the variation in the bond–slip response with the degradation level of CFRP material. In addition, future work may consider the development of a bond–slip model for joints with GFS with and without corrosion damage, based on extended experimental data.

## Figures and Tables

**Table 1:** Material properties of CFRP, steel, and adhesive [22].

Properties	CFRP	Steel	Adhesive (Araldite 420)
Tensile strength (MPa)	2659	539	30
Yield strength (MPa)	N/A	381	N/A
Tensile modulus (GPa)	155.9	219.6	1.744

**Table 2:** Experimental program and specimens.

Group	Specimen Label	Bond Length (mm)	Corrosion Rate of Steel *r*_corr_ g/(m^2^·a)	Calculated Exposure Duration (s)
1	CF30-C0-1,2,3	30	0	0
	CF30-C2-1,2,3		200	3418.07
	CF30-C3-1,2,3		400	6836.14
	CF30-C4-1,2,3		650	11,108.72
	CF30-C5-1,2,3		1500	25,635.51
	CF30-CX-1,2,3		3000	51,271.02
2	GF30-C0-1,2,3	30	0	0
	GF30-C2-1,2,3		200	3418.07
	GF30-C3-1,2,3		400	6836.14
	GF30-C4-1,2,3		650	11,108.72
	GF30-C5-1,2,3		1500	25,635.51
	GF30-CX-1,2,3		3000	51,271.02
3	CF50-C5-1,2,3	50	1500	26,671.29
	CF70-C5-1,2,3	70	1500	27,707.07
	CF90-C5-1,2,3	90	1500	28,742.84
	CF110-C5-1,2,3	110	1500	29,778.62
	CF130-C5-1,2,3	130	1500	30,814.40
	CF150-C5-1,2,3	150	1500	31,850.18

**Table 3:** Categories of corrosivity and corresponding corrosion rates [21].

Category	Corrosivity	Corrosion Rate of Steel *r*_corr_ g/(m^2^·a)
C1	Very Low	*r*_corr_ ≤ 10
C2	Low	10 < *r*_corr_ ≤ 200
C3	Medium	200 < *r*_corr_ ≤ 400
C4	High	400 < *r*_cor_ ≤ 650
C5	Very High	650 < *r*_cor_ ≤ 1500
CX	Extreme	1500 < r_corr_ ≤ 5500

**Table 4:** Experimental results of CFRP–steel double-strap joints.

Group	Specimen Label	Measured Adhesive Thickness t_a_ (mm)	Predicted Corrosion Mass Loss (g)	Measured Corrosion Mass Loss (g)	Ultimate Load P_ult_ (kN)	Averaged Ultimate Load P_ult.ave_ (kN)	Load Reduction (%)	Failure Mode
1	CF30-C0-1	0.39	0	0	50.87	50.32	0	DL
	CF30-C0-2	0.44		0	49.75			DL
	CF30-C0-3	0.48		0	50.35			DL
	CF30-C2-1	0.43	2.97	3.05	48.09	48.67	3.28	DL + ID ^1^
	CF30-C2-2	0.48		2.90	50.26			DL + ID
	CF30-C2-3	0.41		2.65	47.65			DL + ID
	CF30-C3-1	0.45	5.94	5.75	46.44	47.52	5.56	DL + ID
	CF30-C3-2	0.42		5.8	48.09			DL + ID
	CF30-C3-3	0.44		5.85	48.03			DL + ID
	CF30-C4-1	0.41	9.65	9.45	47.63	46.27	8.05	DL + ID
	CF30-C4-2	0.45		9.05	44.92			DL + ID
	CF30-C4-3	0.40		9.65	46.19			DL + ID
	CF30-C5-1	0.42	22.28	22.30	43.31	42.73	15.08	ID + DL ^2^
	CF30-C5-2	0.42		22.25	42.68			ID + DL
	CF30-C5-3	0.39		22.25	42.20			ID + DL
	CF30-CX-1	0.42	44.55	43.05	42.03	42.01	16.51	ID + DL
	CF30-CX-2	0.48		44.75	41.31			ID + DL
	CF30-CX-3	0.50		44.55	42.70			ID + DL
2	GF30-C0-1	0.43	0	0	49.23	49.22	0	DL + GFS fragments
	GF30-C0-2	0.42		0	50.28			DL + GFS fragments
	GF30-C0-3	0.45		0	48.16			DL + GFS fragments
	GF30-C2-1	0.48	2.97	2.90	48.11	47.90	2.68	DL + ID
	GF30-C2-2	0.42		2.65	48.75			DL + ID
	GF30-C2-3	0.50		2.95	46.85			DL+ID
	GF30-C3-1	0.42	5.94	5.85	48.21	47.74	3.01	DL + ID
	GF30-C3-2	0.47		5.85	47.55			DL + ID
	GF30-C3-3	0.39		5.90	47.47			DL + ID
	GF30-C4-1	0.41	9.65	9.75	46.37	47.58	3.33	DL + ID
	GF30-C4-2	0.48		9.80	48.54			DL + ID
	GF30-C4-3	0.46		9.60	47.83			DL + ID
	GF30-C5-1	0.41	22.28	24.15	45.47	46.26	6.01	DL + ID
	GF30-C5-2	0.49		22.15	46.17			DL + ID
	GF30-C5-3	0.46		22.35	47.13			DL + ID
	GF30-CX-1	0.42	44.55	44.10	43.12	42.57	13.51	ID + DL
	GF30-CX-2	0.39		46.25	42.23			ID + DL
	GF30-CX-3	0.46		44.35	42.35			ID + DL
3	CF50-C5-1	0.49	23.18	23.15	64.01	63.15	13.86	DL + ID
	CF50-C5-2	0.48		23.15	63.55			DL + ID
	CF50-C5-3	0.42		23.15	61.88			DL + ID
	CF70-C5-1	0.45	24.08	24.35	75.65	73.13	20.91	DL + ID
	CF70-C5-2	0.46		24.50	73.31			DL + ID
	CF70-C5-3	0.35		24.40	70.44			DL + ID
	CF90-C5-1	0.50	24.98	25.25	80.96	83.67	20.89	DL + ID
	CF90-C5-2	0.48		25.25	86.02			DL + ID
	CF90-C5-3	0.39		25.05	84.02			DL + ID
	CF110-C5-1	0.45	25.88	26.40	90.08	86.52	24.48	DL + ID
	CF110-C5-2	0.40		26.25	82.17			DL + ID
	CF110-C5-3	0.44		26.05	87.30			DL + ID
	CF130-C5-1	0.46	26.78	27.15	86.65	87.46	27.27	DL + ID
	CF130-C5-2	0.41		27.25	87.50			DL + ID
	CF130-C5-3	0.48		27.35	88.23			DL + ID
	CF150-C5-1	0.44	27.68	27.85	87.19	84.73	31.38	DL + ID
	CF150-C5-2	0.48		27.75	81.62			DL + ID
	CF150-C5-3	0.43		27.75	85.39			DL + ID

Notes: ^1.^ DL represents CFRP delamination, and ID represents steel–adhesive interface debonding. ^2.^ Mixed failure modes are sorted by proportion: ID + DL means that the area of steel–adhesive interface debonding is greater than the area of CFRP delamination; DL + ID means that the area of CFRP delamination is greater than the area of steel–adhesive interface debonding.

## Data Availability

The original contributions presented in this study are included in the article. Further inquiries can be directed to the corresponding author.

## References

[1] Global Wind Energy Council (2024). Global Offshore Wind Report 2024 EB/OL.

[2] Delzendeh Moghadam M., Fathi A., Chaallal O. (2024). Retrofitting of steel structures with CFRP: Literature review and research needs. Appl. Sci..

[3] Borrie D., Al-Saadi S., Zhao X.-L., Raman R.S., Bai Y. (2021). Bonded CFRP/steel systems, remedies of bond degradation and behaviour of CFRP repaired steel: An overview. Polymers.

[4] Kupski J., De Freitas S.T. (2021). Design of adhesively bonded lap joints with laminated CFRP adherends: Review, challenges and new opportunities for aerospace structures. Compos. Struct..

[5] Karthikeyan N., Naveen J. (2025). Progress in adhesive-bonded composite joints: A comprehensive review. J. Reinf. Plast. Compos..

[6] Li C., Ke L., He J., Chen Z., Jiao Y. (2019). Effects of mechanical properties of adhesive and CFRP on the bond behavior in CFRP-strengthened steel structures. Compos. Struct..

[7] Wu C., Zhao X., Duan W.H., Al-Mahaidi R. (2012). Bond characteristics between ultra high modulus CFRP laminates and steel. Thin-Walled Struct..

[8] Fernando D., Yu T., Teng J.G. (2014). Behavior of CFRP laminates bonded to a steel substrate using a ductile adhesive. J. Compos. Constr..

[9] Al-Mosawe A., Al-Mahaidi R., Zhao X.L. (2016). Bond behaviour between CFRP laminates and steel members under different loading rates. Compos. Struct..

[10] Al-Zubaidy H., Al-Mahaidi R., Zhao X.L. (2012). Experimental investigation of bond characteristics between CFRP fabrics and steel plate joints under impact tensile loads. Compos. Struct..

[11] Doroudi Y., Fernando D., Zhou H., Nguyen V.T., Ghafoori E. (2020). Fatigue behavior of FRP-to-steel bonded interface: An experimental study with a damage plasticity model. Int. J. Fatigue.

[12] Yu Q.Q., Gao R.X., Gu X.L., Zhao X.L., Chen T. (2018). Bond behavior of CFRP-steel double-lap joints exposed to marine atmosphere and fatigue loading. Eng. Struct..

[13] Borrie D., Liu H.B., Zhao X.L., Raman R.S., Bai Y. (2015). Bond durability of fatigued CFRP-steel double-lap joints pre-exposed to marine environment. Compos. Struct..

[14] Galvez P., Abenojar J., Martinez M.A. (2019). Effect of moisture and temperature on the thermal and mechanical properties of a ductile epoxy adhesive for use in steel structures reinforced with CFRP. Compos. Part B Eng..

[15] Heshmati M., Haghani R., Al-Emrani M. (2017). Durability of CFRP/steel joints under cyclic wet-dry and freeze-thaw conditions. Compos. Part B Eng..

[16] Li A., Xu S., Wang H., Zhang H., Wang Y. (2019). Bond behaviour between CFRP plates and corroded steel plates. Compos. Struct..

[17] Zhang P., Li H., Liu Y., Tao X., Qi Y., Sheikh S.A. (2025). Interfacial bonding performance of CFRP sheet reinforced corroded steel plates under fatigue loading. Constr. Build. Mater..

[18] Xie J., Yang M., Kainuma S. (2023). estigation on irreversible deterioration mechanism of CFRP–steel adhesive joint based on the hydrothermal aging behaviour of resin matrix. Structures.

[19] Viana G.M., Costa M., Banea M.D., da Silva L.F. (2017). A review on the temperature and moisture degradation of adhesive joints. Proc. Inst. Mech. Eng. Part L J. Mater. Des. Appl..

[20] Torres-Acosta A.A. (2002). Galvanic corrosion of steel in contact with carbon-polymer composites. I: Experiments in mortar. J. Compos. Constr..

[21] (2012). Corrosion of Metals and Alloys—Corrosivity of Atmospheres—Classification, Determination and Estimation.

[22] Wu C., Yu Y.Z., Tam L.H., Orr J., He L. (2021). Effect of glass fiber sheet in adhesive on the bond and galvanic corrosion behaviours of CFRP-Steel bonded system. Compos. Struct..

[23] (2008). Standard Test Method for Tensile Properties of Polymer Matrix Composite Materials.

[24] (2016). Standard Test Methods for Tension Testing of Metallic Materials.

[25] (2014). Standard Test Method for Tensile Properties of Plastics.

[26] Zhao X.L., Zhang L. (2007). State-of-the-art review on FRP strengthened steel structures. Eng. Struct..

[27] Ren X., Sherif M.M., Wei Y., Lyu Y., Sun Y., Ozbulut O.E. (2022). Effect of corrosion on the tensile and fatigue performance of CFRP strand Sheet/Steel double strap joints. Eng. Struct..

[28] Batuwitage C., Fawzia S., Thambiratnam D., Al-Mahaidi R. (2017). Durability of CFRP strengthened steel plate double-strap joints in accelerated corrosion environments. Compos. Struct..

[29] Kim Y.J., Bumadian I., Park J.S. (2016). Galvanic current influencing interface deterioration of CFRP bonded to a steel substrate. J. Mater. Civ. Eng..

[30] Kim Y.J., Bumadian I. (2016). Electrochemical reactions for steel beams strengthened with CFRP sheets. Eng. Struct..

[31] Batuwitage C., Fawzia S., Thambiratnam D., Al-Mahaidi R. (2017). Evaluation of bond properties of degraded CFRP-strengthened double strap joints. Compos. Struct..

[32] Peng F. (2005). Research on the Interfacial Performance of Metallic Structures Strengthened with Fibre Reinforced Polymers. Ph.D. Thesis.

[33] Mariam M., Afendi M., Majid M.A., Ridzuan M.J., Azmi A.I., Sultan M.T. (2019). Influence of hydrothermal ageing on the mechanical properties of an adhesively bonded joint with different adherends. Compos. Part B Eng..

[34] Yu Y.Z., Tam L.H., Wu C. (2023). A universal solution of the bond behaviour between CFRP-steel double strap joints considering steel yielding. Compos. Struct..

[35] Pang Y.Y., Wu G., Wang H.T., Su Z.L., He X.Y. (2020). Experimental study on the bond behavior of the CFRP-steel interface under the freeze–thaw cycles. J. Compos. Mater..

[36] Pang Y., Wu G., Wang H., Gao D., Zhang P. (2021). Bond-slip model of the CFRP-steel interface with the CFRP delamination failure. Compos. Struct..

[37] Bocciarelli M., Colombi P., Fava G., Poggi C. (2009). Prediction of debonding strength of tensile steel/CFRP joints using fracture mechanics and stress based criteria. Eng. Fract. Mech..

